# Looking after young eye patients in hospital

**Published:** 2010-03

**Authors:** Caroline Ayilo, Dianne Pickering, Fay Gallant, Ingrid Mason

**Affiliations:** Paediatric nurse, Gertrude's Garden Children's Hospital, Kenya. **cayilo@gerties.org**; Registered General Nurse, Norfolk and Norwich University Hospital, UK. **dianne.pickering@nnuh.nhs.uk**; Registered Paediatric Nurse. James Paget Hospital, Norfolk, UK.; CBM Capacity Development Officer and Medical Advisor, PO Box 58004, 00200 City Square, Ring Road Parklands, Nairobi, Kenya.

**Figure:**
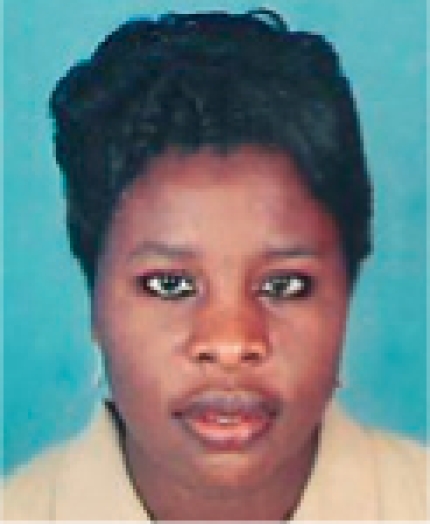


**Figure:**
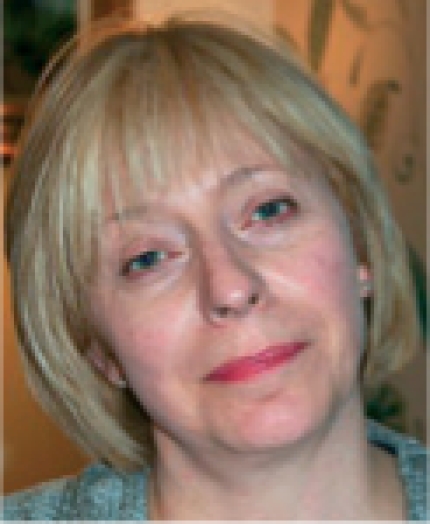


**Figure:**
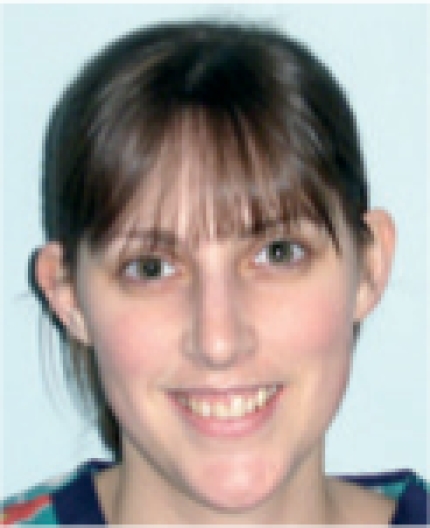


**Figure:**
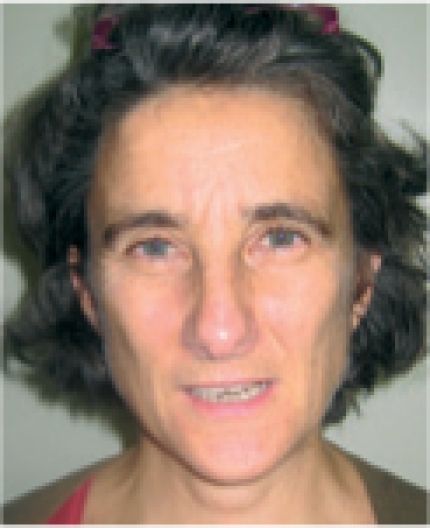


Admission into hospital can be an anxious and unsettling time for a child, whether it is for a planned eye procedure or as a result of an eye injury. There is a lot you can do to make the hospital experience as positive as possible to reduce stress and anxiety for both child and parent.

## The physical environment

It is advisable to have a dedicated ward for children. It should be bright, clean, and colourful. You can use simple drawings to decorate the walls and ceilings. Colourful curtains and bedspreads will also brighten up the ward.

If a child is visually impaired, it is necessary to make the area around them as free from obstacles as possible. Checking that the child is properly acquainted with their surroundings will help to ensure that they are safe and feel more confident. Painting doors, windows, door handles, and sign boards in contrasting colours will help a child with low vision navigate more easily and feel more at ease in a new environment.

## Friendly conduct

It is important to be kind and to show your concern for the children in your care. Spend time with children and talk to them in a language they can understand. By listening to children and treating them with respect, you build their confidence and make it possible for them to voice their fears or concerns.

When talking to children, it is important to try and sit next to them or to crouch down so that your head is at approximately the same height as theirs. This will help you to avoid ‘talking down’ at them, which children can find intimidating.

Children like being called by their name or nickname - you can ask the parents what name the child prefers.

## Keeping parents and children together

Allow parents or carers to stay with their children as much as possible. In most hospitals, the only place a parent is not allowed to enter is the operating theatre.

If a child needs to stay overnight, strongly encourage parents to stay with them. This will support the child's recovery and reduce anxiety and pain.

Where possible, conduct all treatments (apart from operations) with the parents present. The nurse or doctor can describe the treatment in simple terms to the parent before it begins. The parent can then explain this to the child in a manner that the child will understand.

The parent or guardian should hold the child during treatments or interventions as this reduces the stress of the child and makes it easier for the doctor or nurse to work quickly and efficiently.

## The importance of play

Play is very important to children. Do what you can to support and encourage children to play. A play area should be available for children and this can usually be provided, even if the area is small. Allow children to play with their siblings (under supervision), provided that it will not affect their recovery.

Play can also be used to alleviate children's fear and prepare them for their stay in hospital - some hospitals even have dedicated ‘play specialists’ to work with children.

Encourage parents to role play with their child to win their confidence. For example, parents can let the nurse pretend to instil eye drops into their own eye; this will show the child that it is safe and will help them know what to expect.

Distraction is also a helpful technique. For example, getting children to focus their attention on a toy or a game before a blood test can help to reduce their anxiety and pain.

## Admission to hospital

- As already mentioned, children should ideally have their own ward. If that is not possible, place the child in a bed close to the nurses’ station.
- Welcome the parent and child, show them where their bed is, and explain where everything is on the ward.
- Give the parent a name band with the child's name (and nickname, if necessary) to put on the child's arm.
- The child does not need to wear hospital clothes unless they are going to the operating theatre, but clean clothes are advisable.
- Favourite toys or games can be brought in by the parents.
- Identify any food preferences. Depending on the hospital policy, parents can bring in food for their child.

**Figure:**
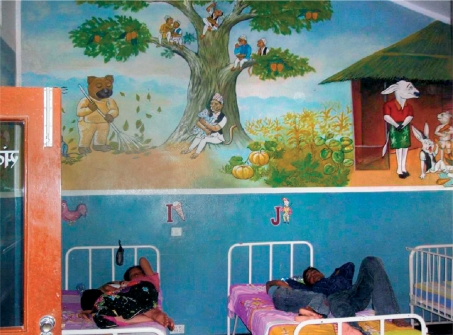
A child-friendly ward. NEPAL

Lumbini Eye Institute, Nepal

**Figure:**
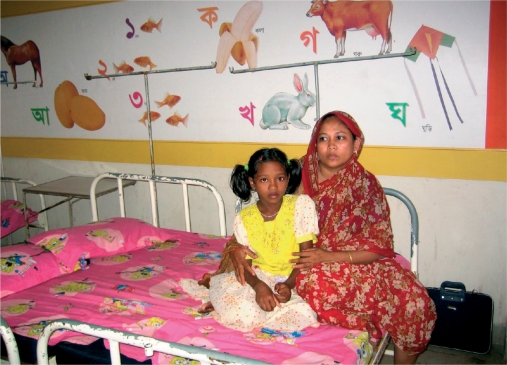
It is important to keep children and parents together. BANGLADESH

Clare Gilbert

## The operation

- The ophthalmologist should explain the procedure, the process, and the prognosis to the parent away from the child. The parents will then be able explain this in a way their child can understand. It is important that the child knows what is going to happen to them as this helps to build trust.
- If the child is to have a general anaesthetic, it is important to make sure the parents understand that their child must not have any solid food for at least six hours before the operation. Babies can be breast fed up to four hours before the operation, but babies who have formula milk must not be fed for at least six hours before the operation. Babies and children can and should drink water during this time, but must stop at least two hours before the operation.
- Encourage the parent or carer to refer to the anaesthetic as a ‘special sleep’. Explain that, upon waking, the child's eye may be sore for a short while and that their eye may be covered by a special patch. Covering a favourite toy's eye with an eye patch may also be helpful.
- Ideally, the parent should accompany their child from the ward as far as the operating theatre. If the child is to have a general anaesthetic, given in the anaesthetic room, then the parent can stay with the child until he or she is ‘asleep’. Parents are not allowed inside the operating theatre.
- Explain to the parents that a child who has had a general anaesthetic may feel nauseous for the next 24 hours. Parents should encourage, but not force, their child to drink. As long as the child is drinking, it does not matter if they do not want to eat for a few days.

## Postoperative care

- Encourage the parents to put their child's favourite toy or comforter on the bed so it is there when the child returns from the operating theatre. This will help to reduce the child's anxiety.
- Make sure that the parents are with their child when he or she wakes from the eye operation. Parents can accompany - or even carry - their child back to the ward.
- The doctor should talk to the parents away from the child to explain how the operation went, what was found or done, and the likely prognosis. Understanding the prognosis will help parents to comply with instructions for care and use of medication after they return home.
- The parents should explain what is going to happen with the support of a nurse, if required. The nurse can again make use of the toys in the play area to demonstrate to the child how their eye pad will be removed and how the parent will instil their eye medication.

## Going home

When appropriate, nursing staff should show parents the treatment or ongoing care the child is going to need after discharge from the hospital, such as putting on an eye patch, instilling eye drops, or putting in eye ointment (see page 16). Parents should be encouraged to assist with these and to take over doing the procedure when they feel ready. This will make parents more confident and able to look after their child once they leave the hospital.

In most instances, going home after a successful eye operation is a happy occasion. However, if a child has lost their vision, or has a poor visual outcome, they and their parents need additional support. Ensure that the parents understand what additional services are available and encourage them to make use of them, including low vision care and rehabilitation. Parents may need further support, particularly if they are distressed (see box). It is important that parents get help, as the child should not be discharged into a stressed family setting.

In addition, the child (and parent) may have started to feel secure in a hospital environment and may be fearful of going home. Counselling may be required, which will include guidance on the roles of family members once the child is at home.

If a child has become blind, the parents will need guidance on how to communicate with and behave towards their child. This is a very large and important topic which goes beyond the scope of this article; however, the following are some ideas to discuss with parents:

- Always say who you are when you enter the room the child is in.
- Explain what you are doing or are going to do.
- Do not whisper or make noises without explaining what you are doing.
- Encourage the child to explore their environment using their hands, feet, and other senses.

Two helpful books are *Show me what my friends can see* and *Helping children who are blind* - see Useful Resources on page 11.

If there is a counsellor or occupational therapist, refer the child and parent for their advice and support.

## Conclusion

This article gives some suggestions for making the hospital environment less intimidating and more friendly for children and their parents. It should be possible to adapt these ideas to suit your particular work environment, at very little cost. Taking time to consider the needs of both the child and parent in hospital will assist in the child's recovery and raise the profile of the hospital in the eyes of the community.

**Support for parents**

If parents are told bad news (for example, if their child will lose an eye or the sight in one eye) they may react in a number of ways. Some become withdrawn as they take in the information, while others may become aggressive and demanding. Both these reactions are quite normal, and represent a response to stress or grief: the grief of losing the completely healthy child they thought they had.

Be patient and kind, but honest. If parents become distressed, offer to talk to them again later in the day or the following day. Always try to keep these appointments as doing so instils confidence and trust.

It may be advisable to enlist the help of professional counsellors, where available, as they have skills in counselling.

Encourage parents to form self-help groups. Put parents of children with the same problems in contact with each other so that they have someone to talk to who shares their experiences and concerns. These counsellors should help the parents to discover ways to tell the child. A counsellor can be present with the parents when the child is being told. This takes time and should not be rushed.

